# Reduction of Pain Sensitivity After Somatosensory Therapy in Adults with Cerebral Palsy

**DOI:** 10.3389/fnhum.2013.00276

**Published:** 2013-06-24

**Authors:** Inmaculada Riquelme, Anna Zamorano, Pedro Montoya

**Affiliations:** ^1^Research Institute on Health Sciences, University of the Balearic Islands, Palma de Mallorca, Spain; ^2^Department of Nursing and Physiotherapy, University of the Balearic Islands, Palma de Mallorca, Spain

**Keywords:** somatosensory therapy, cerebral palsy, sensitivity, pain, somatosensory processing

## Abstract

**Objective:** Pain and deficits in somatosensory processing seem to play a relevant role in cerebral palsy (CP). Rehabilitation techniques based on neuroplasticity mechanisms may induce powerful changes in the organization of the primary somatosensory cortex and have been proved to reduce levels of pain and discomfort in neurological pathologies. However, little is known about the efficacy of such interventions for pain sensitivity in CP individuals.

**Methods:** Adults with CP participated in the study and were randomly assigned to the intervention (*n* = 17) or the control group (*n* = 20). The intervention group received a somatosensory therapy including four types of exercises (touch, proprioception, vibration, and stereognosis). All participants were asked to continue their standardized motor therapy during the study period. Several somatosensory (pain and touch thresholds, stereognosis, proprioception, texture recognition) and motor parameters (fine motor skills) were assessed before, immediately after and 3 months after the therapy (follow-up).

**Results:** Participants of the intervention group showed a significant reduction on pain sensitivity after treatment and at follow-up after 3 months, whereas participants in the control group displayed increasing pain sensitivity over time. No improvements were found on touch sensitivity, proprioception, texture recognition, or fine motor skills.

**Conclusion:** Data suggest the possibility that somatosensory therapy was effective in eliciting changes in central somatosensory processing. This hypothesis may have implications for future neuromodulatory treatment of pain complaints in children and adults with CP.

## Introduction

Cerebral palsy (CP) may lead not only to motor disability but also to somatosensory deficits. Recent neuroimaging studies have provided evidence of significant alterations in white matter fibers connecting to sensory cortex (radiata and internal capsule), indicating that CP injuries might be reflecting disruption of sensory as well as motor connections (Hoon et al., 2002, 2009; Thomas et al., 2005). Accordingly, previous studies have shown that CP individuals display poorer tactile discrimination, stereognosis, and proprioception (McLaughlin et al., 2005; Sanger and Kukke, 2007; Wingert et al., 2009), as well as enhanced pain than healthy controls (Vogtle, 2009; Doralp and Bartlett, 2010; Malone and Vogtle, 2010; Parkinson et al., 2010; Riquelme and Montoya, 2010; Riquelme et al., 2011). Moreover, studies from our lab have proven that reduced touch sensitivity are associated with increased pain sensitivity in children with early brain injury (Riquelme and Montoya, 2010), suggesting a potential link between abnormal somatosensory experiences in early life and long-term changes in pain processing (Schmelzle-Lubiecki et al., 2007). In this sense, animal studies have revealed that brain damage provoked by asphyxia may be worsened by aberrant sensorimotor experience during maturation and could be responsible for the disabling movement disorders observed in children with CP (Coq et al., 2008).

Rehabilitation techniques based on neuroplasticity mechanisms utilizes task specific training and massed practice to drive reorganization and improve sensorimotor function (Taub et al., 1999). It is known that intensive training of somatosensory stimulation, as it occurs in musicians, may induce powerful changes in the organization of the primary somatosensory cortex (Pantev et al., 2001; Schaefer et al., 2005). Somatosensory and sensoriomotor therapies including repetitive touch stimulation, two-point discrimination training, stretching exercises, and posture training have been also proved to reduce levels of pain and discomfort in neurological pathologies such as amputees, complex regional pain syndrome, and somatic tinnitus (Flor et al., 2001; Latifpour et al., 2009; Moseley and Wiech, 2009).

In the present work, we conducted a randomized controlled study to examine the influence of a 12 weeks somatosensory stimulation therapy on pain (pain pressure thresholds), touch sensitivity (tactile threshold, stereognosis, texture recognition), proprioception, and fine motor skills in adults with CP. According with previous studies, we hypothesized that intensive training of somatosensory processing (including repetitive touch stimulation, stereognostic exercises, touch discrimination, and proprioception) would result in reduced pain sensitivity in persons with CP.

## Materials and Methods

### Participants

Subjects with CP were recruited from occupational centers established in Majorca and Albacete (Spain) between January 2010 and July of 2011. Potential subjects were initially identified by their own physicians and invited to participate using an informational letter explaining the details of the research study. Inclusion criteria were: (1) age between 18 and 40 years old, (2) absence of chronic pain (defined as persistent and generalized pain for more than 6 months), and (3) cognitive level that allows understanding and participating in the therapy activities. Augmentative communication devices and information from parents and caregivers were used as needed to facilitate data collection in subjects with communication difficulties.

Forty adults with CP met the inclusion criteria and decided to participate in the study. They were randomly assigned to one of two study groups: intervention (*n* = 20) (six females; mean age = 30.16, SD = 4.78) or control (*n* = 20; seven females; mean age = 31.15, SD = 4.86). Participants in the control group were in the waiting list for this intervention, and they were aware that there was another condition receiving a somatosensory training. At the moment of the study, all participants were receiving a standardized physical therapy with an emphasis on maintenance of motor skills (postural control, balance, range of motion, gait, etc.), and they were asked to continue with it during the study period. Three participants of the intervention group interrupted the study after the second session and five control participants did not attend to the follow-up assessment. Type of CP and cognitive level were obtained from health records. Level of gross motor impairment was determined using the Gross Motor Function Classification Scale (GMFCS) (Palisano et al., 1997) and level of fine motor impairment was determined using the Manual Ability Classification System (MACS) (Eliasson et al., 2006). Table 1 displays clinical characteristics of both groups.

**Table 1:** **Clinical characteristics of individuals with cerebral palsy**.

ID	Group	Sex	Age	CP subgroup	GMFCS	MACS	Mental retardation
1	I	F	32	A	1	1	Mild
2	I	M	33	BS	5	4	Mild
3	I	M	40	A	2	1	Mild
4	I	M	27	D	4	4	None
5	I	F	29	BS	2	1	Mild
6	I	F	31	D	4	3	Mild
7	I	M	36	BS	4	3	Mild
8	I	M	31	BS	2	2	None
9	I	M	26	D	4	3	None
10	I	M	35	BS	5	5	None
11	I	M	25	BS	4	4	None
12	I	M	24	A	1	1	Mild
13	I	F	32	BS	2	5	None
14	I	F	25	A	1	1	Mild
15	I	F	24	BS	4	1	Moderate
16	I	M	34	BS	2	4	None
17	I	M	32	BS	2	4	None
18	C	M	31	BS	4	4	Mild
19	C	F	28	BS	4	5	None
20	C	M	30	D	2	2	None
21	C	M	30	A	1	1	Mild
22	C	M	28	A	1	1	None
23	C	M	32	BS	1	1	None
24	C	M	31	BS	4	2	None
25	C	M	22	BS	5	1	None
26	C	F	28	BS	4	1	None
27	C	F	33	BS	4	1	Mild
28	C	F	37	D	1	1	Moderate
29	C	F	22	BS	3	1	None
30	C	M	27	A	1	1	Mild
31	C	M	32	BS	5	3	Mild
32	C	F	24	A	1	1	Mild
33	C	F	31	BS	1	3	Mild
34	C	M	32	BS	2	2	Mild
35	C	M	29	BS	4	3	Mild
36	C	M	32	BS	4	2	None
37	C	M	30	BS	1	4	Moderate

*C, control group; I, intervention group; M, male; F, female; BS, bilateral spastic; US, unilateral spastic; D, dyskinetic; A, ataxic*.

All participants granted written informed consent according with the Declaration of Helsinki. Parents or legal tutors signed informed consents and participants corroborated their decisions to participate in the study. The study was approved by the Ethics Committee of the Regional Government of the Balearic Islands.

### Somatosensory assessment

Several somatosensory and motor parameters were assessed before (pre-test), immediately after (post-test), and 3 months after the therapy (follow-up). Control participants were assessed at the same time as participants from the intervention group. Assessments were performed in the occupational centers by one member of the research team (IR), who was blind for the condition to which participants were allocated and different from physiotherapists providing the intervention. Following outcome measures were obtained.

#### Pressure pain

Pressure pain thresholds (expressed in kgf/cm^2^) were measured with a digital dynamometer and using a flat rubber tip (1 cm^2^). Subjects were asked to say “pain” when the pressure became painful. Pressure was released when either the pain detection threshold had been reached or when the maximum pressure of the algometer was reached. Pressure stimuli were applied bilaterally in pseudo-randomized order at six body locations (lips, cheeks, thenar eminences, thumb fingers, index fingers, and both hand dorsum) until three measurements at each location were obtained. Two average pain threshold scores were computed considering measurements at the FACE (lips, cheeks) and HANDS (thenar eminences, thumb fingers, index fingers, and both hand dorsum). Subjects were familiarized with the assessment procedure by using non-painful ranges to relieve potential anxiety. The reliability of this procedure for assessing pain sensitivity has been demonstrated in previous studies (Cathcart and Pritchard, 2006).

#### Touch

Fine touch sensitivity by using von Frey monofilaments (Keizer et al., 2008) was measured bilaterally at the same six body locations described before. The test consisted of a set with plastic filaments of different diameter (0.14–1.01 mm). The assessment was performed by touching the skin in a perpendicular way, pressing it slowly down until it buckled, holding it steady during 1.5 s and removing it in the same way as it was applied. After several practice trials, subjects were instructed to notify if they felt any sensation of touch by saying “yes” or “not.” The procedure started with a thick filament and depending on subjects’ answers, thicker or thinner filaments were applied. The sensitivity score for each body location was calculated as the mean of the three thinnest filaments detected. Null stimuli were also used to find false positive responses and responses delayed more than 3 s were noted as abnormal. Body locations were stimulated in a pseudo-randomized order. Two average tactile threshold scores were computed considering body locations at the FACE (lips, cheeks) and HANDS (thenar eminences, thumb fingers, index fingers, and both hand dorsum).

#### Texture recognition

Participants were touched bilaterally on cheek, lip, and hand by using objects with different textures (soft, hard, smooth, and rough). Participants wore a sleeping mask and they were asked if the stimulus was soft or hard (smooth or rough) to facilitate the answer. The four texture sensations were tested, giving one point for each correct answer. Texture recognition has been used frequently as a way to test sensitivity (Carey and Matyas, 2005).

#### Stereognosis

Ten common objects were used (coin, bank note, scissors, pencil, pen, comb, towel, sponge, glass, and cup) to assess stereognosis of both hands. Participants wore a sleeping mask and they were instructed to touch the object with one hand and to identify it. For individuals with motor difficulties, the examiner moved the object in participants’ hands. Stereognosis was scored from 0 to 2 for each object (2 = normal, the object was correctly identified; 1 = impaired, participant was able to describe some features of the object; 0 = absent, participant was unable to identify the object) and a sum score was computed. This procedure was adapted from the Nottingham Sensory Assessment test, whose reliability has been proven in previous studies (Gaubert and Mockett, 2000).

#### Proprioceptive tasks

Proprioception was assessed by asking participants to reproduce or to describe passive joint movements (wrist, elbow, metacarpophalangeal joints from the second to the fifth digit, and metacarpophalangeal joint of thumb) performed by the experimenter with participants wearing a sleeping mask. Proprioception was scored according to following criteria: 2 = Normal, able to achieve final joint position within 10° range of error; 1 = partially impaired, able to appreciate joint movement but fail to detect movement direction; 0 = impaired, no appreciation of joint movement. This procedure was adapted from the Nottingham Sensory Assessment test, whose reliability has been proven in previous studies (Gaubert and Mockett, 2000).

#### Fine motor skills

The Purdue Pegboard Test was used to assess fine motor skills of the hand. During the test, the subject was seated in front of a pegboard with two cups on the far-right and far-left corner each containing 25 pins. The task consisted in picking up one pin at a time from the cup (left or right, depending on which hand is used) by using the thumb and index finger only and placing it on the appropriate row (left or right). Subjects were instructed to place as many pins as possible in 30 s. Three trials were performed: one with the right, one with the left, and one with both hands. For the trial with both hands, subjects were instructed to pick up simultaneously one pin with the right hand and one pin with the left hand, and to place them on the corresponding row. The assembly part of the original test was excluded. This test has been previously used to assess fine hand performance in individuals with CP (Arnould et al., 2007).

### Somatosensory therapy

The somatosensory therapy consisted of two 45-min weekly sessions for 12 weeks (24 sessions) and conducted by two trained physiotherapists. At the beginning of the study, all participants were already receiving one or two sessions per week of physiotherapy at their occupational centers. Participants were asked to continue with their scheduled sessions in agreement with their therapists.

The somatosensory therapy included four types of somatosensory tasks focused on face and hands: touch (e.g., touching different textures, tactile location), proprioception (e.g., pushing and weight exercises), vibration (e.g., massage at different frequencies), and stereognosis (e.g., recognition of geometric forms and common household objects). Task difficulty was increased from the first to the second weekly session. Physiotherapists were instructed to document all clinical observations within each session.

### Statistical analysis

Analyses of variance (ANOVAs) were computed on dependent variables by using GROUP (intervention vs. control) as between-subject factor, and TIME (pre-test vs. post-test vs. follow-up) and BODY LOCATION (face vs. hand) as within-subject factors. Significant interaction effects were further analyzed by using *post hoc* pairwise mean comparisons provided by the ANOVA procedure in SPSS.

## Results

Participants in the intervention and the control group were similar in age, gross motor performance, manual ability, touch sensitivity, pain thresholds, stereognosis, proprioception, texture recognition, and fine motor performance scores at the beginning of the study.

**Figure 1:**
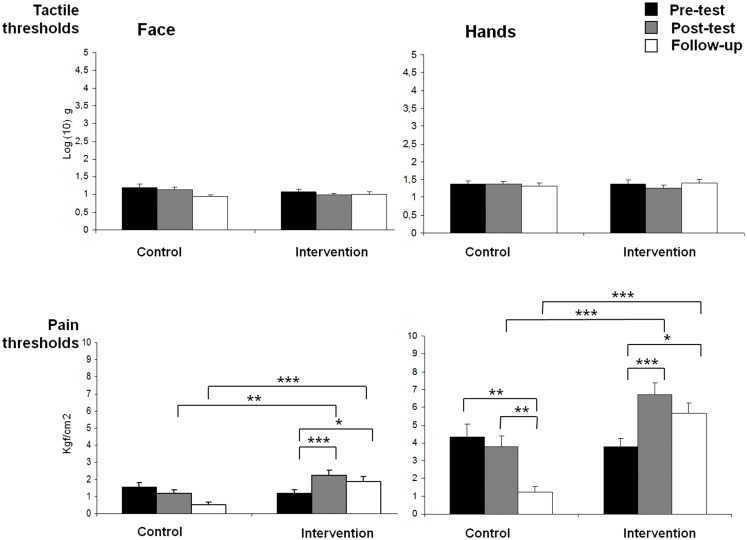
Means of pain and tactile thresholds for each group (control and intervention), assessment time (pre-test, post-test, and follow-up) and body side (face and hands) (*p < 0.05, **p < 0.01, ***p < 0.001).

Figure 1 shows means and standard deviations of tactile and pain thresholds on face and hands for both groups during the three assessment intervals (pre-test, post-test, and follow-up). A significant GROUP × TIME × BODY interaction effect was found on pain sensitivity [*F*(2,29) = 3.63, *p* < 0.05], indicating that participants in the intervention group displayed higher pain thresholds (reduced sensitivity) on both body locations during post-test and follow-up assessments than controls (all *post hoc* pairwise mean comparisons were significant at *p* < 0.01). Moreover, *post hoc* comparisons revealed that pain thresholds on both body locations were significantly increased from pre- to post-test (*p*s < 0.001) and from pre-test to follow-up (*p*s < 0.05) in the intervention group. By contrast, *post hoc* comparisons also indicated that pain thresholds on hand were significantly reduced (increased sensitivity) from pre-test to follow-up (*p* < 0.01), and from post-test to follow-up (*p* < 0.01) in the control group. In addition, significant effects due to GROUP [*F*(1,30) = 22.18, *p* < 0.001] (intervention group > control group), TIME [*F*(2,60) = 7.29, *p* < 0.01] (post-test > pre-test and post-test > follow-up), BODY LOCATION [*F*(1,30) = 621.84, *p* < 0.001] (hands > face), GROUP × TIME [*F*(2,60) = 15.05, *p* < 0.001], and GROUP × BODY LOCATION [*F*(1,30) = 10.39, *p* < 0.01] were observed.

For tactile thresholds, a significant GROUP × TIME effect was found [*F*(2,29) = 3.72, *p* < 0.05], showing a reduction of tactile thresholds from post-test to follow-up assessments in the control group (*p* < 0.05), but no significant effects in the intervention group. In addition, a significant effect due to BODY LOCATION was yielded [*F*(1,30) = 48.05, *p* < 0.001], indicating that tactile thresholds were higher on hands than on face.

**Figure 2:**
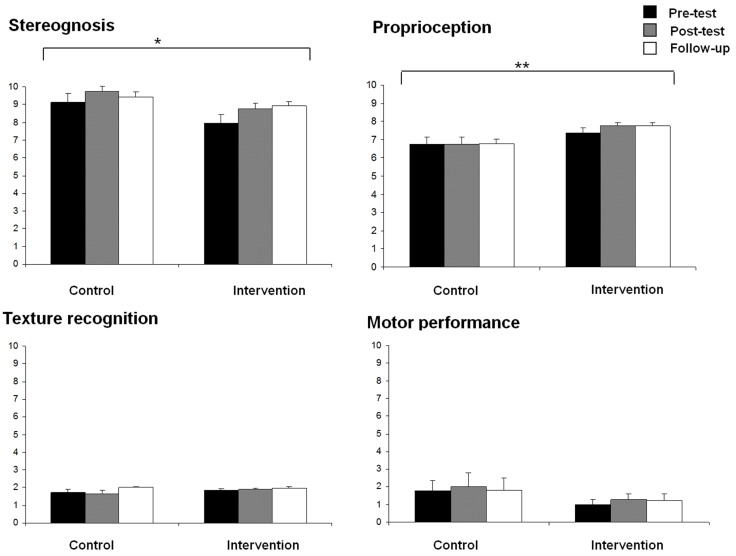
Means of stereognosis, proprioception, texture recognition, and motor performance for each group (control and intervention) at all assessment times (pre-test, post-test, and follow-up) (*p < 0.05, **p < 0.01).

Stereognosis and proprioception scores for both groups at the three assessment times (pre-test, post-test, and follow-up) are displayed in Figure 2. Significant effects due to GROUP [*F*(1,27) = 4.89, *p* < 0.05] and TIME [*F*(2,26) = 5.46, *p* < 0.05] were found on stereognosis, showing more reduced scores in the intervention group than in the control group, and an increased stereognosis from pre- to post-test assessment. No GROUP × TIME interaction effect was obtained.

For proprioception scores, a significant effect due to GROUP [*F*(1,27) = 5.04, *p* < 0.05] was also found, showing better proprioception in the intervention group than in the control group. No other significant effects were observed.

No significant effects were found on fine motor function and texture recognition scores (Figure 2).

## Discussion

The objective of this study was to evaluate the effects of a somatosensory therapy on pain and touch thresholds, stereognosis, texture recognition, proprioception, and fine motor function in adults with CP. Our results revealed that participants in the intervention group showed a significant reduction of pain sensitivity after treatment, whereas participants in the control group displayed increasing pain sensitivity over time. These changes remained even at follow-up after 3 months. By contrast, no significant improvements in touch thresholds, texture recognition, proprioception, or fine motor skills were observed in any of the groups.

These results are relevant because pain is considered an important comorbidity factor in persons with CP (Vogtle, 2009; Doralp and Bartlett, 2010; Malone and Vogtle, 2010; Parkinson et al., 2010). In previous studies, we have found that individuals with CP reported more pain and lower touch sensitivity than healthy controls, and that clinical pain ratings in CP were associated with reduced touch sensitivity (Riquelme and Montoya, 2010). The present study further revealed that training of somatosensory processing may reduce pressure pain sensitivity in CP. All these findings are in accordance with growing evidence indicating that patients with enhanced pain sensitivity (such as chronic pain patients) are less able to identify the location and characteristics of a tactile stimulus when delivered to a painful body area (Moriwaki and Yuge, 1999; Maihofner et al., 2006). Furthermore, it has been observed that training in discriminating tactile stimuli (Moseley et al., 2008) and graded sensorimotor exercises (Pleger et al., 2005) can reduce pain perception. Although the neurobiological mechanism responsible for this link between sensory training and pain is still unknown, it has been suggested that changes in somatosensory processing could be mediated by changes in primary sensory cortices (cortical reorganization) in response to hyperstimulation (Jenkins et al., 1990; Kattenstroth et al., 2012). Thus, the finding that our somatosensory therapy led to significant reductions in pain sensitivity over time in CP individuals may suggest the possibility that these changes were due to relevant changes in central somatosensory processing. In this sense, previous data have shown that somatosensory therapies are able to reduce pain and discomfort, as well cortical reorganization in amputees, chronic pain, and somatic tinnitus (Flor et al., 2001; Latifpour et al., 2009; Moseley and Wiech, 2009; Moseley and Flor, 2012). Furthermore, it seems that those somatosensory interventions in which patients are required to discriminate actively between the type and location of tactile stimuli may be more effective than mere repetitive and passive body stimulation in reducing pain (Moseley et al., 2008). Thus, it seems plausible that the active components of our intervention might be responsible for the observed pain sensitivity effects in the present study.

Nevertheless, the finding that our somatosensory intervention was able to change pain, but not touch thresholds or somatosensory perception was puzzling. At a glance, this seems contrary to previous studies showing that tactile discrimination training and repetitive stimulation of the body can improve tactile function in healthy individuals (Godde et al., 1996) and patients with chronic pain (Pleger et al., 2005; Moseley et al., 2008). One possible explanation of these contradictory findings could be methodological differences between the present and the rest of studies. Thus, stimulation paradigms in previous studies mainly consisted of repetitive and intensive stimulation at specific sites of the body during minutes or hours, and changes of tactile acuity were often measured by using two-point discrimination thresholds, neither of which occurred here. In the present study, we used more ecological and functional, although less intensive exercises than the mere repetitive body stimulation used in previous studies. Moreover, it has been suggested that different cutaneous mechanoreceptive afferent systems are involved in distinct and separate central systems for processing of somatosensory information (form and texture perception, motion, vibration, stretching) (Johnson, 2001). Here, we used mechanical thresholds (von Frey monofilaments) to test the effects of the somatosensory intervention. Thus, it could be that our assessment tools were not appropriate for measuring the effects of our somatosensory intervention program on touch processing.

Our results also add new evidence about the benefits of somatosensory and sensoriomotor training in CP individuals. Several studies have reported that pressure splints improved the range of movement, balance, dynamic stability, motor control, postural and muscle readiness and walking function, and elicited an enhancement of SEP amplitudes (Hylton and Allen, 1997; Semenova, 1997; Kerem et al., 2001). Furthermore, it has been reported that sensorimotor exercises (e.g., vestibular system activities, balance and postural responses, coordination, motor planning, right-left discrimination training, visual spatial perception, sensory inputs, body awareness) produced an improvement of tactile perception, kinesthesia, graphesthesia, and daily living activities (Bumin and Kayihan, 2001). Again, these changes in sensoriomotor parameters could be attributed to brain mechanism of plasticity elicited by training and practice. In this sense, it is known that sensorimotor integration is based on feedforward and feedback contributions between different areas of somatosensory and motor cortices (Rizzolatti and Luppino, 2001). Thus, it may be argued that the joint activation of both cortical regions during these therapies could modulate the exchange of information between the somatosensory and motor systems, resulting in an improvement of somatosensory processing and motor abilities. In the present study, we found that somatosensory therapy was effective in eliciting long-lasting changes only on pain sensitivity, but not on fine motor skills. Although we have no clear-cut explanation for these results, the fact that individuals of both groups were simultaneously involved in a standardized motor therapy may explain the lack of differential effects on fine motor skills.

The present study has some limitations that should be taken into account for the interpretation of the results. Although individuals with CP seem to be representative of their community, a relative low sample of subject with heterogeneous etiologies was recruited. Moreover, an interference effect individuals’ motor therapy with our intervention protocol could not be discarded. An active control condition, in which non-specific somatosensory stimulation were applied, could have added information to the specificity of our therapy.

All these findings highlight the importance of somatosensory experience in the enhanced sensibility to pain demonstrated in persons with CP (Riquelme and Montoya, 2010). The increase of somatosensory experiences provided by our somatosensory therapy may have effects on pain processing and may reduce pain perception in CP individuals. This hypothesis may have implications for future neuromodulatory treatment of pain complaints in children and adults with CP. Early interventions should be address to decrease sensitivity to pain throughout the adult years.

## Conflict of Interest Statement

The authors declare that the research was conducted in the absence of any commercial or financial relationships that could be construed as a potential conflict of interest.
